# Gemcitabine with a specific conformal 3D 5FU radiochemotherapy technique is safe and effective in the definitive management of locally advanced pancreatic cancer

**DOI:** 10.1038/sj.bjc.6603900

**Published:** 2007-07-24

**Authors:** D Goldstein, G Van Hazel, E Walpole, C Underhill, D Kotasek, M Michael, J Shapiro, T Davies, W Reece, J Harvey, N Spry

**Affiliations:** 1 Department of Medical Oncology, Prince of Wales Hospital, Barker Street, Randwick, New South Wales 2031, Australia; 2 Department of Medical Oncology, Sir Charles Gairdner Hospital, Hospital Avenue, Nedlands, Western Australia 6009, Australia; 3 Department of Medical Oncology, Princess Alexandra Hospital, Mater QRI, Brisbane, Queensland 4101, Australia; 4 Border Medical Oncology, Murray Valley Private Hospital, Nordsvan Drive, Wodonga, 3690 Victoria, Australia; 5 Department of Medical Oncology, Ashford Cancer Centre, Marleston Avenue, Ashford, South Australia 5035, Australia; 6 Department of Medical Oncology, Peter MacCallum Cancer Centre, St Andrews Place, East Melbourne, Victoria 3002, Australia; 7 Department of Medical Oncology, Alfred Hospital, The Alfred, Commercial Road, Prahran, Victoria 3181, Australia; 8 Eli Lilly Australia, 112 Wharf Road, West Ryde 2114, New South Wales, Australia; 9 Department of Radiation Oncology, Princess Alexandra Hospital, Ipswich Road, Woolloongabba, Queensland 4102, Australia; 10 Department of Radiation Oncology, Sir Charles Gairdner Hospital, Hospital Avenue, Nedlands, Western Australia 6009, Australia

**Keywords:** locally advanced, conformal radiation, pancreas cancer, gemcitabine

## Abstract

The aim of this phase II study was to assess the feasibility and efficacy of a specific three-dimensional conformal radiotherapy technique with concurrent continuous infusion of 5-fluorouracil (CI 5FU) sandwiched between gemcitabine chemotherapy in patients with locally advanced pancreatic cancer. Patients with inoperable cancer in the pancreatic head or body without metastases were given gemcitabine at 1000 mg m^−2^ weekly for 3 weeks followed by a 1-week rest and a 6-week period of radiotherapy and concurrent CI 5FU (200 mg m^−2^ day^−1^). The defined target volume was treated to 54 Gy in 30 daily fractions of 1.8 Gy. After 4 weeks' rest, gemcitabine treatment was re-initiated for three cycles (days 1, 8, 15, q28). Forty-one patients were enrolled. At the end of radiotherapy, one patient (2.4%) had a complete response and four patients (9.6%) had a partial response; at the end of treatment, three patients (7.3%) had a complete response and two patients (4.9%) had a partial response. Median survival time was 11.7 months, median time to progression was 7.1 months, and median time to failure of local control was 11.9 months. The 1- and 2-year survival rates were 46.3 and 9.8%, respectively. Treatment-related grade 3 and 4 toxicities were reported by 16 (39.0%) and four (9.8%) patients, respectively. Sixteen out of 41 patients did not complete the planned treatment and nine due to disease progression. This approach to treatment of locally advanced pancreatic cancer is safe and promising, with good local control for a substantial proportion of patients, and merits testing in a randomised trial.

Pancreatic cancer is characterised by a tendency for both metastatic spread and local failure; it is one of the most difficult cancers to treat and has a 5-year survival rate of 4% (Jemal *et al*, 2005). Surgical resection gives the best chance for a possible cure; however, only 10–20% of patients present with potentially resectable pancreatic cancer, and most of these patients will ultimately succumb to recurrent or metastatic disease. The majority of patients with pancreatic cancer present with locally advanced or metastatic disease that is inoperable (Cardenes *et al*, 2006).

Chemotherapy as a sole modality improves survival in patients with locally advanced or metastatic disease (Palmer *et al*, 1994; Glimelius *et al*, 1996); however, even with the current regimens, median overall survival has ranged from 3.3 to 11.5 months in phase II trials and has generally been not more than 7 months in phase III trials (Burris *et al*, 1997; Berlin *et al*, 2002; Colucci *et al*, 2002; Maisey *et al*, 2002; Moore *et al*, 2003; Petty *et al*, 2003; Rocha Lima *et al*, 2004; Lopes and Rocha Lima, 2005; Louvet *et al*, 2005; Oettle *et al*, 2005). There is little published information on the effectiveness of chemotherapy alone in preventing local failure, but an apparent improvement in survival has been reported in two recent large phase III studies, which had a significant subset of patients with locally advanced disease (Cunningham *et al*, 2005; Poplin *et al*, 2006a). The median survival times in the locally advanced groups were 9.9 months (J Neoptolemos, personal communication) and 9.1 months (Poplin *et al*, 2006b).

The best survival times for inoperable locally advanced disease have been in response to combined chemotherapy and radiotherapy with survival times ranging from 8 to 11 months in studies conducted in the 1980s, and from 8 to 14.5 months in more recent studies (Dickler and Abrams, 2005; Wilkowski *et al*, 2005). The delivery of adequate doses of radiation to the pancreas is limited by the sensitivity of the normal structures nearby including the kidneys, liver, stomach, small intestine, and spinal cord. Specific three-dimensional conformal radiotherapy (3D CRT) techniques employ computed tomography (CT) planning to design radiation beams that conform much more closely to the shape of the tumour than is possible with the older two-dimensional techniques, allowing a higher dose to be delivered to the tumour while confining the dose to the surrounding healthy tissue to within safe and tolerable levels (Emami *et al*, 1991).

There is no common approach to the management of locally advanced pancreatic cancer in Australia and New Zealand. Multimodal treatment approaches are the most likely avenues for further development. The aim of this open-label phase II study was to determine the toxicity and efficacy of protocol-specific 3D CRT integrated with modern chemotherapy in the treatment of locoregional pancreatic cancer, including toxicity attributable to late effects of radiotherapy. Two independent cohorts of patients in Australia were studied; we report here on the locally advanced cohort, where surgical resection was not possible and the aim of the treatment was to obtain prolonged local control. Further analysis of the entire study population (including quality of life) will be reported elsewhere.

## MATERIALS AND METHODS

### Eligibility criteria

Eligible patients for this prospective phase II study (B9E-AY-S168) had a histological or cytological diagnosis of pancreatic adenocarcinoma in the head or body of the pancreas, with metastatic disease excluded on whole body CT series, and locoregional staging of the primary disease undertaken with dual-phase spiral CT. Other inclusion criteria included ECOG performance status of 0–2; adequate bone marrow function (white blood cell count ⩾3.5 × 10^9^ l^−1^, absolute neutrophil count (ANC) ⩾1.5 × 10^9^ l^−1^, platelets ⩾100 × 10^9^ l^−1^, and haemoglobin ⩾10.0 g dl^−1^); and serum creatinine ⩽150 *μ*mol l^−1^. Exclusion criteria included prior cytotoxic chemotherapy; significant loss of body weight (e.g., >15% weight loss since surgery or diagnosis); and previous abdominal radiotherapy. The study was approved by the human Ethics Review Boards of the participating institutions and conducted according to ICH Good Clinical Practice Guidelines, including obtaining written informed consent. Two studies were undertaken in parallel, investigating this treatment programme as both definitive therapy for locally advanced inoperable patients and adjuvant therapy in the management of high-risk resected patients. To be eligible for the locally advanced arm, all patients must have been assessed by a surgeon specialising in the upper gastrointestinal tract and considered inoperable or strongly declined surgery. At the time of initiating this study, it was not the policy nor belief of surgeons in Australia that downstaging was a feasible strategy, and it is the practice of most surgeons to undertake exploration of patients considered to be borderline operable. No patients who were offered surgery, who then refused, were reported by the investigators as a reason for recruitment for this trial.

### Study design

**Figure 1:**
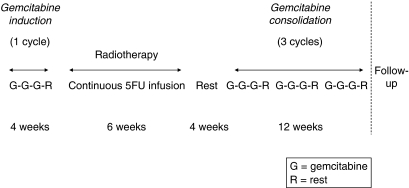
Study design.

One cycle of gemcitabine chemotherapy was planned, followed by radiotherapy and concomitant CI 5FU, 4 weeks of rest, and an additional three cycles of gemcitabine chemotherapy (Figure 1). Each cycle consisted of gemcitabine (1000 mg m^−2^) administered weekly for 3 weeks followed by a week of rest. Gemcitabine was given by intravenous infusion approximately for 30 min. Patients were followed up every 3 months for 2 years. A full blood count was performed on days 1, 8, and 15 of the chemotherapy cycles and weekly during radiotherapy.

Follow-up after completion of the treatment was every 2 months. This included physical examination, full blood count, liver function, and CA 19-9 levels. CT scans were done on the basis of either clinical suspicion on the part of the investigator or three successive rises in CA 19-9 levels.

### Gemcitabine treatment

To start the cycle, the ANC had to be ⩾1.5 × 10^9^ l^−1^ and platelets ⩾100 × 10^9^ l^−1^. Dose adjustments for haematologic toxicity were based on the neutrophil and platelet counts on the day of administration. The dose of gemcitabine was reduced by 25 or 50%, respectively, if the ANC was 1.25–1.499 × 10^9^ or 1.0–1.249 × 10^9^ l^−1^, or if platelets were 75–99.999 × 10^9^ or 50–74.999 × 10^9^ l^−1^. The gemcitabine dose was withheld in the case of ANC <0.999 × 10^9^ l^−1^ or platelets <50 × 10^9^ l^−1^; once the neutrophil count was ⩾1.5 × 10^9^ l^−1^ and the platelet count ⩾100 × 10^9^ l^−1^, subsequent treatment was delivered at 75% of the previous dose. If non-haematological CTC grade 3 or 4 toxicity occurred (excluding alopecia, nausea, or vomiting), treatment was withheld until toxicity returned to, or was better than, grade 1. Subsequent treatment was 75% of the previous dosage. The induction period was not allowed to exceed 5 weeks; deferred weekly cycles were missed rather than extending the induction treatment period.

### 5FU infusion

5FU was given by continuous intravenous infusion. A starting dose of 200 mg m^−2^ day^−1^, 7 days a week, was given, beginning on the first day of radiation therapy and continuing until the completion of radiation treatment. The infusion was discontinued for at least 7 days in the case of a fall in ECOG performance status by one class or greater; weight loss >10% over the duration of treatment; or any of the following grade 3 or 4 symptoms: nausea or vomiting not controlled by maximal antiemetic therapy; stomatitis; diarrhoea; hand-foot syndrome; ANC <1.0 × 10^9^ l^−1^ or platelet count <50 × 10^9^ l^−1^; and febrile neutropaenia. Resumption of 5FU (with a 25% dose reduction) was not started until toxicity receded to ⩽grade 1. Missed weeks of 5FU infusion were not made up, to avoid prolonging the radiochemotherapy.

### Radiotherapy

Patients were immobilised using a reliable set up apparatus, and required to be supine with arms over head during simulation and treatment. Planning CT scan thickness needed to be no thicker than 5 mm and not greater than 5 mm intervals to minimise the extent of volume averaging and provide good tumour and normal tissue imaging. Oral contrast was recommended to be seen in the duodenal loop and intravenous contrast to assist definition of the major abdominal arterial structures. The planned target volume was the gross tumour volume +1 cm and the dose prescription was 54 Gy in 30 daily fractions of 1.8 Gy in accordance with International Commission on Radiation Units (ICRU) 50 principles (ICRU, 1993). An additional superior inferior margin was added for respiratory movement. Normal tissue tolerance dose constraints for kidney, liver, and spinal cord were as defined in Table 1 (Emami *et al*, 1991). Volume extension to incorporate ‘at-risk’ nodal sites or ‘normal pancreas’ was discouraged because node prophylaxis in this setting has not yet been shown to be beneficial, the main site of failure continues to be local mass site and because radiotherapy-induced toxicity increases with treatment volume size. Field arrangement commonly employed four axially placed fields, although non-coplanar techniques could be employed too (and were used in 42.5% of patients) (Osborne *et al*, 2006).

Discontinuation of radiotherapy for at least 7 days was required for a fall in performance status by one ECOG class or greater below ECOG 1, or for grade 3 or 4 toxicity of any of the following: nausea or vomiting was not controlled by maximal antiemetic therapy; diarrhoea; platelet count <50 × 10^9^ l^−1^; and weight loss of >10%. Resumption of radiotherapy was not allowed until the weight loss or fall in performance status was reversed, or the toxicities had receded to ⩽grade 2, regardless of time missed (except for haematological toxicity, when it could be recommenced when the platelet count reached ⩾50 × 10^9^ l^−1^). It was intended that all radiotherapy be completed within 8 weeks, including treatment interruptions. Treatment with radiotherapy took priority over 5FU infusions: if both treatments were ceased, then reinstitution of radiotherapy occurred before reinstitution of 5FU infusion, according to the outlined guidelines.

### Efficacy criteria

Objective response was assessed by comparison of pre-treatment and restaging CT scans (not <4 weeks apart). The products of the two largest dimensions for individual lesions were compared using the following definitions. Complete response: disappearance of all known disease with tumour markers within the normal range. Partial response: ⩾50% decrease in total tumour size with no appearance of new lesions or progression of any lesion. Stable disease: <50% decrease in total tumour size up to a 25% increase in the size of one or more indicator lesions. Progressive disease: ⩾25% increase in the size of at least one indicator lesion or the appearance of new lesions. A responder was defined as a patient with complete or partial response. Prospective studies have demonstrated the potential value of changes in CA 19-9 level with treatment (Maisey *et al*, 2005; Ferrone *et al*, 2006). An increase of 15% in serum CA 19-9 over the previous level on two consecutive occasions 3 weeks apart was considered to suggest progression and mandate additional investigation by CT scan. Confirmation of response by consecutive CT scans was not required.

Time-to-event analyses using the Kaplan–Meier method were calculated from the start of therapy and were not adjusted for second-line therapies. Survival time was calculated to the date of death and censored on date last known alive. Time to progression was calculated to the date of progression or death and censored on date last known alive. Failure of local control was calculated to the date of progression in the pancreas and censored on the date of last scan, if the patient died or completed follow-up without evidence of local progression. Local progression was defined as ⩾25% increase in the size of a lesion in the pancreas or the appearance of a new lesion in the pancreas.

### Quality assurance for radiotherapy

The participating radiation oncologist was provided with a planning scan data set and invited to plan a virtual patient. The planning review was coordinated through the Sir Charles Gairdner Hospital in accordance with RTOG/EORTC (Radiation Therapy Oncology Group/European Organisation for Research and Treatment of Cancer) procedures. This was undertaken at each site, before the first enrolled patient, to ensure consistent protocol compliance and correct unintentional ambiguities. Major technique violations were identified in two cases: in one case, the whole pancreas was included in the planning target volume although it had been the intention of the protocol to include only the gross tumour plus a margin, and in the second case, an excessive margin was employed inferiorly in the post-operative scenario (greater than the margins in other directions). Ambiguities in the protocol were identified and corrected, and the replans were acceptable. A central review of the plans of patients selected at random underwent quality assurance by a subgroup of radiation oncologists, which is currently in manuscript preparation. Late toxicity data were prospectively collected for hepatic and renal adverse events up to 6 months post-therapy, which is approximately 10 months post-radiotherapy, and cause of death was investigated retrospectively if either major renal or hepatic function was involved in the cause of death.

### Statistical methods

Exact 95% confidence intervals (CI) are reported. Dose intensity was calculated from the date of first infusion to the last planned day of the last cycle the patient received drug. Since the planned dose of gemcitabine was 1000 mg m^−2^ week^−1^ for 3 weeks and then 1 week off in a 4-week cycle, the planned dose intensity was 750 mg m^−2^ week^−1^, which takes into account the planned week off and then accounts for any additional delays. SAS v8.2 was used.

## RESULTS

### Baseline characteristics

**Figure 2:**
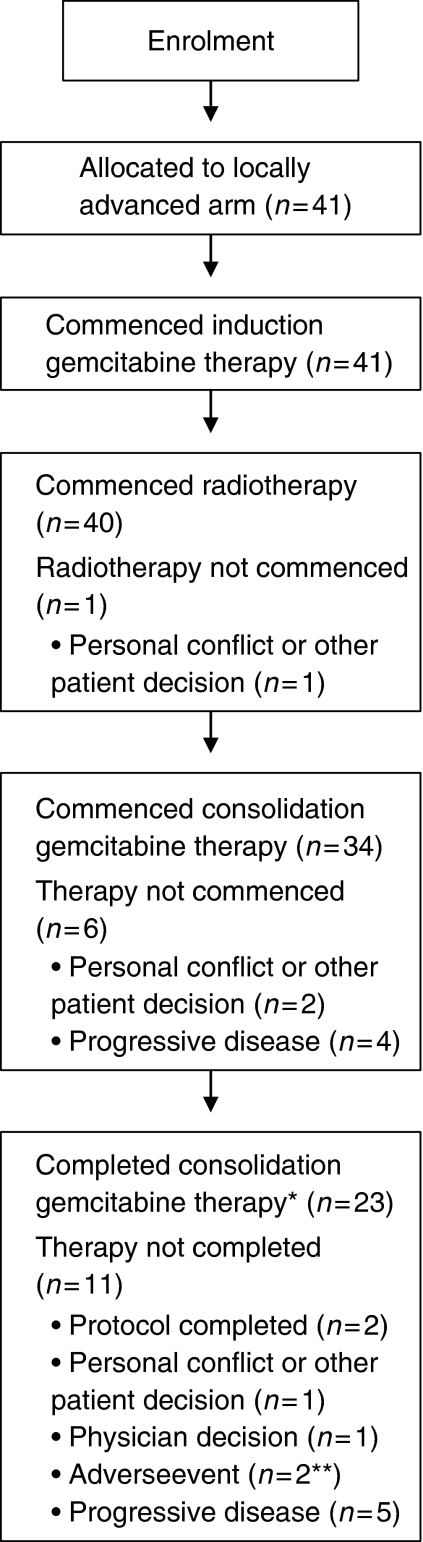
Patient disposition. ^*^A patient was considered to have completed consolidation gemcitabine therapy if he received three gemcitabine infusions in the third consolidation cycle. ^**^One patient had abdominal pain, the other leukopaenia.

The baseline characteristics of the 41 patients enrolled in the locally advanced arm of the study are shown in Table 2. Patient attrition is described in Figure 2.

### Efficacy

At the end of radiotherapy, one patient (2.4%) had a complete response and four patients (9.8%) a partial response, for an overall response rate of 12.2% (Table 3). At the end of consolidation chemotherapy, three patients (7.3%) had a complete response and two patients (4.9%) had a partial response, for an overall response rate of 12.2%. Twenty-four patients (58.5%) had stable disease at the end of radiotherapy and nine patients (22.0%) at the end of chemotherapy. Four patients did not start consolidation chemotherapy because of progressive disease (Figure 2); progression was reported in liver (one patient), lung (one), and lymph nodes (two). Five patients did not complete consolidation chemotherapy because of progressive disease: ascites (one); progression in pancreas (one), liver (one), both pancreas and liver (one), and site not reported (one).

**Figure 3:**
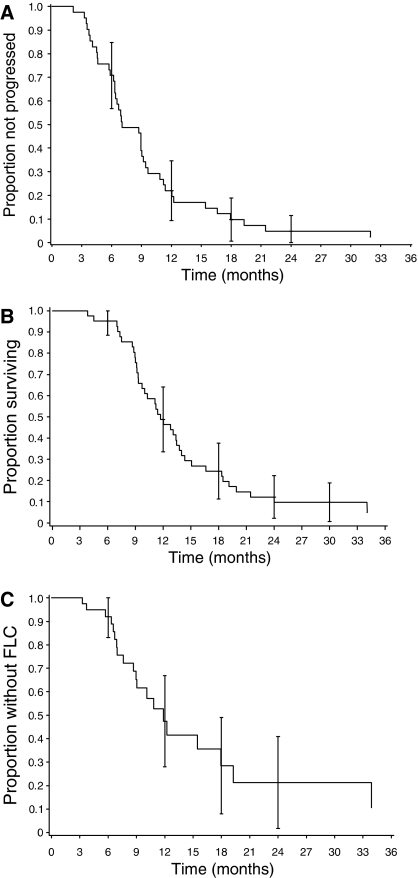
Time-to-event measures. (**A**) Time to progression. (**B**) Survival time. (**C**) Time to failure of local control^*^ (FLC). ^*^Failure of local control was calculated to the date of progression in the pancreas, and was censored on the date of last scan if the patient died or completed follow-up without evidence of local progression, even if the patient progressed systemically.

Figure 3 shows the Kaplan–Meier curves for time to progression and survival. Median time to progression was 7.1 months (95% CI: 6.3, 9.2 months) and median survival time was 11.7 months (95% CI: 9.7, 13.7 months). The 1- and 2-year survival rates were 46.3% (95% CI: 31.1, 61.6%) and 9.8% (95% CI: 0.7, 18.8%), respectively.

For the 32 patients who progressed and the first site of progression could be assessed, 10 patients first progressed locally (i.e., progression or a new lesion in the pancreas), 27 patients first progressed systemically, and five patients first progressed both locally and systemically. Five patients with systemic progression subsequently also had documented local progression. The most common site of first systemic progression was liver (12 patients) and then lung (six patients). Twenty patients (48.8%) failed local control, 20 patients (48.8%) progressed or died without evidence of failing local control, and one patient (2.4%) did not progress. The median time to failure of local control was 11.9 months (95% CI: 8.9, 17.9 months). Figure 3 shows the Kaplan–Meier curve for time to failure of local control.

### Safety

Twenty-three patients (56.1%) reported at least one grade 3 toxicity and seven patients (17.1%) at least one grade 4 toxicity (Table 4). Treatment-related grade 3 and 4 toxicities were reported by 16 (39.0%) and four (9.8%) patients, respectively. The majority of these were haematologic, of which three patients reported grade 4 events, and grade 4 febrile neutropaenia was reported by one patient. Treatment-related gastrointestinal toxicities occurred in five patients (12.2%) with no grade 4 episodes. Two patients experienced grade 4 cholangitis (one during initial chemotherapy and one during radiation) and two patients had biliary stent obstruction (one during radiation and one post-radiation). Two patients had subacute bowel obstruction (one during radiation and one post-radiation), which resolved with conservative management. There were no episodes of treatment-related ischaemic heart disease or hand-foot syndrome.

Clinical complications of haematological toxicity were rare: grade 3/4 infection/febrile neutropaenia was reported by three patients (7.3%). One patient had radiation recall syndrome. Five patients required a transfusion of packed red blood cells and one patient a transfusion of platelets.

During radiotherapy and the following rest period, grade 3/4 haematological toxicity was experienced by 5.0% of patients, and symptomatic gastrointestinal nausea and vomiting by 10.0 and 5.0% of patients, respectively. Subsequent problems with gastric outlet obstruction were observed in one patient and no patient required new biliary stenting. Toxicity attributable to late effects of radiotherapy was not seen to affect liver, bowel, or spinal cord. One patient experienced renal failure in the follow-up period. This patient had end-stage metastatic liver disease with normal renal function 2 weeks prior; the renal failure was therefore considered secondary to the progressive disease and neither the radiation nor chemotherapy treatment.

Haematological toxicity occurred mostly during the gemcitabine-alone phases, with grade 3/4 neutropaenia in both the gemcitabine treatment period before (9.8%) and after (26.5%) radiotherapy, and grade 3/4 thrombocytopaenia in the gemcitabine treatment period after radiotherapy (8.8%).

### Dose delays and reductions

More patients required a delay or reduction in the post-radiotherapy gemcitabine doses (30 out of 34 patients, 88.2%) than in pre-radiotherapy gemcitabine (18 out of 41 patients, 43.9%). Approximately, 60% of patients were able to receive three cycles of gemcitabine post-radiotherapy (25 out of 41 patients). A dose adjustment in 5FU was required for 20.0% of patients (eight out of 40 patients) and in radiotherapy for 20.0% (eight out of 40 patients). Sixteen out of 41 patients did not complete the planned treatment. This was due to progressive disease for nine out of 41 patients (22.0%); the other seven patients (17.1%) did not complete therapy because of adverse events (two patients), personal conflict or other patient decision (two patients – two because of toxicity), and physician's decision for treatment effects (one patient).

### Dose intensity

The planned dose intensity for gemcitabine (pre- and post-radiotherapy) was 750 mg m^−2^ week^−1^. The mean achieved and relative dose intensity of gemcitabine was higher in the gemcitabine treatment period before radiotherapy (656.7 mg m^−2^ week^−1^, 87.6%, respectively) than afterwards (505.5 mg m^−2^ week^−1^, 67.4%). The planned dose intensity of 5FU was 1400 mg m^−2^ week^−1^ and the mean achieved dose intensity was 1409.1 mg m^−2^ week^−1^ (100.7%). The planned dose intensity of radiotherapy was 54 Gy over 6 weeks and the mean achieved dose intensity was 52.0 Gy (96.2%).

## DISCUSSION

There are three pertinent findings from our study: (i) protocol-specific 3D CRT with sandwich gemcitabine chemotherapy has acceptable acute toxicity; (ii) this regimen was not associated with major late toxicity to adjacent organs; and (iii) the efficacy end points were encouraging with favorable survival and local control in this population of patients with locally advanced pancreatic cancer.

Haematological toxicity, nausea, and vomiting were modest during radiochemotherapy and the rest period afterwards, and the radiochemotherapy did not appear to significantly compromise systemic treatment with gemcitabine in the later months of therapy. For the majority of patients, not completing the planned therapy was attributable to disease progression or personal decisions, not to treatment-related effects. While there were more reductions and delays in gemcitabine in the consolidation period than in the induction period, and the dose intensity was therefore lower in the consolidation period, it was not unduly compromised, and the majority of patients (60%) were able to receive the planned three cycles of gemcitabine post-radiotherapy. It should be noted that the rules for dose adjustments were such that if the gemcitabine dose was reduced on day 8 or 15 of a cycle, then the dose would not be re-escalated on day 1 of the next cycle, which may have contributed to the lower dose intensity as the trial progressed. Significant late toxicity affecting kidney, bowel, liver, or spinal cord function was not observed – and these concerns were prospectively assessed – nor were they observed in our parallel post-operative study of 22 patients (reported elsewhere). In addition, there was only one case of subsequent gastric outlet obstruction and no biliary obstruction. Since we are not reporting later follow-up at this time, we cannot exclude any very late effects that may yet occur, but this data regarding the safety of our approach are encouraging.

The median survival time for patients in this study was 11.7 months, which, along with the 1-year survival rate of 46.3%, compares favourably with other contemporary CRT phase II studies (Rich *et al*, 2004; Wilkowski *et al*, 2005; Willett *et al*, 2005). We did not record whether any second-line chemotherapies were administered, and we cannot exclude a small contribution to our survival data from such therapy. In contrast to CRT, chemotherapy alone has appeared to be associated with inferior survival times in two recent phase III studies that examined both locally advanced and metastatic populations. Poplin *et al* (2006a) reported overall median survival times of 4.96, 6.01, and 6.47 months in patients receiving standard gemcitabine treatment, fixed dose rate gemcitabine and gemcitabine–oxaliplatin, respectively; median survival in the locally advanced group was 9.1 months (Poplin *et al*, 2006b). Cunningham *et al* (2005) reported median survivals of 6 and 7.4 months in patients receiving gemcitabine and gemcitabine-capecitabine, respectively; median survival was 9.9 months in the locally advanced group (J Neoptolemos, personal communication). A recent small randomised study comparing gemcitabine alone with additional combined concurrent cisplatin and 5FU with external beam radiation for locally advanced disease has been reported in abstract form (Chauffert *et al*, 2006). An exceptional median survival of 14.3 months was reported for the gemcitabine-alone arm. In contrast, the outcome for the combined therapy arm was a significantly poorer median survival of 8.4 months, which is also inferior to the other combined series reported above. Explanations for this remain speculative in the absence of detailed descriptions of the radiotherapy technique and quality assurance measures employed, but the size of the disparity in outcome does suggest that the choice of regimens and the radiation technique and schedule are vital to achieve optimal outcomes.

Good local control was observed in the patients on whom follow-up data was available, with a local failure rate of 48.8% and median time to failure of local control of 11.9 months. Since local progression is often characterised by severe, difficult-to-control neuropathic pain, we see this outcome as an important measure of efficacy of this approach. Older studies of radiochemotherapy in patients with locally advanced pancreatic cancer have had local failure rates of 58, 72, and 78% (Whittington *et al*, 1984; Gastrointestinal Tumor Study Group, 1985; Roldan *et al*, 1988). More recent studies of radiochemotherapy have tended not to report duration of local control but rather first site of relapse, with local relapse being uncommon as the first site in treated patients (Blackstock *et al*, 2003), which makes comparison and interpretation of the potential impact of radiotherapy more difficult.

The baseline characteristics of the cohort indicate that this study population is typical of patients with locally advanced pancreatic cancer, and, in particular, sex, age, and performance status were not more favourable than those reported in other recent studies (Blackstock *et al*, 2003; Rich *et al*, 2004).

The optimum scheduling of chemotherapy and CRT is not clear. One approach is to focus on inducing downstaging and subsequent operability (Wilkowski *et al*, 2006). The impact of inducing surgery in initially inoperable patients remains experimental. Our focus has been on optimising outcomes in the absence of any further surgery. The use of initial chemotherapy allows identification of rapidly progressing patients who do not then undergo prolonged inappropriate therapy, and may also delay the emergence of systemic disease, enhancing the benefit of local control (Moureau-Zabotto *et al*, 2006; Rana *et al*, 2006; Huguet *et al*, 2007). Despite our finding of some impact on the post-radiotherapy dose intensity and the significant loss of patients due to disease progression or personal decisions during treatment, current chemotherapy agents do not have a high enough response rate for the CRT to be confidently delayed until the completion of a standard 6-month chemotherapy programme, considering the significant problems associated with local progression in tumours that fail to respond to chemotherapy. Delaying radiotherapy until month 4 has been proposed on the basis of a recent retrospective outcome analysis (Huguet *et al*, 2007) and seems to be a pragmatic compromise until the emerging novel targeted agents can be shown to have contributed sufficiently to allow reconsideration of radiotherapy and chemotherapy scheduling.

We believe that we have demonstrated that protocol-specific 3D radiochemotherapy with initial and subsequent systemic gemcitabine is tolerable, feasible, and effective, and offers good local control for a substantial proportion of patients with locally advanced disease. This regimen merits further evaluation as a foundation for adding novel targeted agents. An optimised combined regimen should then be studied in a randomised trial against systemic therapy alone in patients with locally advanced pancreatic cancer.

## Figures and Tables

**Table 1:** Tissue dose tolerance TD 5/5 irradiation of one-third and two-thirds of target volume

**Target volume**	**TD 5/5 irradiation of one-third of target volume (Gy)**	**TD 5/5 for irradiation of two-thirds of target volume (Gy)**
Stomach	60	55
Small intestine	50	Not established
Kidney	50	30
Liver	50	35
Spinal cord	50	50

Tolerance dose (TD) for various organs for one-third and two-thirds of target volume at the 5% complication rate 5 years after treatment.

Taken from Emami *et al* (1991).

**Table 2:** Baseline patient characteristics

**Characteristic**	***N*=41**
*Sex*	15 (36.6%)
M	26 (63.4%)
F	
Age (mean±s.d.)	63.9±10.8
	
Age (range)	30–79
	
*ECOG performance status* a	
0	17 (41.5%)
1	21 (51.2%)
2	2 (4.9%)
	
*T stage*	
T1	4 (9.8%)
T2	12 (29.3%)
T3	16 (39.0%)
T4	8 (19.5%)
TX	1 (2.4%)
	
*N stage*	
N0	25 (61.0%)
N1	10 (24.4%)
NX	6 (14.6%)

ECOG, Eastern Cooperative Oncology Group.

a The ECOG performance status of one patient was unknown.

**Table 3:** Summary of response by visit

	**End RT (*N*=41)**	**End CT (*N*=41)**
Complete response	1 (2.4%)	3 (7.3%)
Partial response	4 (9.8%)	2 (4.9%)
Stable disease	24 (58.5%)	9 (22.0%)
Progressive disease	6 (14.6%)	15 (36.6%)
Unknown	2 (4.9%)	2 (4.9%)
Not done^a^	4 (9.8%)^b^	10 (24.4%)^c^

End CT, end of gemcitabine consolidation chemotherapy; End RT, end of radiotherapy.

a Reasons not done:

b Moved to another hospital (1), hospitalised with bowel obstruction (1), patient decision (2).

c Moved to another hospital (1), patient decision (2+1 due to toxicity), patient admitted to hospital (1), deteriorating health (1), site error (2), scan done too late (1), no reason given (1).

**Table 4:** CTC grade 3 and 4 toxicities

	**Regardless of causality**		**Possibly related to therapy**	
**CTC Group**	**Grade 3 (*N*=41)**	**Grade 4 (*N*=41)**	**Grade 3 (*N*=41)**	**Grade 4 (*N*=41)**
Overall	23 (56.1%)	7 (17.1%)	16 (39.0%)	4 (9.8%)
				
*Blood/bone marrow*	14 (34.1%)	3 (7.3%)	12 (29.3%)	3 (7.3%)
Haemoglobin	1 (2.4%)	1 (2.4%)	1 (2.4%)	1 (2.4%)
Leukocytes	9 (22.0%)	1 (2.4%)	9 (22.0%)	1 (2.4%)
Neutrophils/granulocytes	10 (24.4%)	2 (4.9%)	8 (19.5%)	2 (4.9%)
Platelets	3 (7.3%)	0	3 (7.3%)	0
				
*Gastrointestinal*	13 (31.7%)	2 (4.9%)	5 (12.2%)	0
Anorexia	1 (2.4%)	0	1 (2.4%)	0
Ascites	1 (2.4%)	0	0	0
Constipation	4 (9.8%)	0	1 (2.4%)	0
Dehydration	2 (4.9%)	0	2 (4.9%)	0
Diarrhoea without colostomy	1 (2.4%)	0	0	0
Dyspepsia/heartburn	1 (2.4%)	0	0	0
Gastritis	1 (2.4%)	0	0	0
Nausea	5 (12.2%)	0	3 (7.3%)	0
Vomiting	3 (7.3%)	0	2 (4.9%)	0
Other gastrointestinal	4 (9.8%)	2 (4.9%)	0	0
				
*Hepatic*	3 (7.3%)	2 (4.9%)	1 (2.4%)	0
Alkaline phosphatase	4 (9.8%)	0	0	0
Bilirubin	1 (2.4%)	1 (2.4%)	0	0
GGT	1 (2.4%)	0	0	0
AST	2 (4.9%)	1 (2.4%)	1 (2.4%)	0
ALT	1 (2.4%)	1 (2.4%)	0	0
Infection/febrile neutropaenia	2 (4.9%)	1 (2.4%)	0	1 (2.4%)
				
*Metabolic/laboratory*	8 (19.5%)	2 (4.9%)	0	0
Hyperglycaemia	8 (19.5%)	2 (4.9%)	0	0
Pain	7 (17.1%)	0	1 (2.4%)	0

ALT, alanine aminotransferase; AST, aspartate aminotransferase; GGT, *γ*-glutamyl transpeptidase.

Patients who reported more than one toxicity are counted more than once in this table. Relatedness should be treated with caution because some study sites interpreted ‘therapy’ to mean only gemcitabine.

Other categories (regardless of causality): cardiovascular (general) (*n*=5): hypertension (two patients), thrombosis (2), and other cardiovascular/general (1); constitutional symptoms (*n*=2): fatigue (1) and weight loss (1); endocrine (*n*=1): other endocrine (1); haemorrhage (*n*=1): melena/gastrointestinal bleeding (1); neurology (*n*=2): CNS cerebrovascular ischaemia (1) and mood alteration-depression (1); pulmonary (*n*=3): dyspnea (1) and pulmonary-other (2); renal/genitourinary (*n*=1): urinary frequency/urgency (1). All of these were grade 3 toxicities.
